# Paradoxical Embolism in a Patient with Aortic Valve Endocarditis: A Case Report

**DOI:** 10.7759/cureus.2301

**Published:** 2018-03-10

**Authors:** Eduardo L Santos, Andre D Lima, Luca Dompieri, Arthur C Holanda, Maria A Aquino, Renato D Lopes

**Affiliations:** 1 Department of Cardiology, Federal University of Pernambuco, Recife, BRA; 2 Medical School, Federal University of Pernambuco, Recife, BRA; 3 Division of Cardiology, Duke University Medical Center

**Keywords:** infective endocarditis, aortic valve endocarditis, paradoxical embolism, endocarditis

## Abstract

This report describes a case of aortic valve endocarditis with systemic and paradoxical pulmonary embolism in a patient with congenital interventricular communication. The patient underwent cardiac surgery and did not have a favorable outcome, presenting refractory cardiogenic shock and subsequently dying while in the hospital. This is an extremely rare case of paradoxical embolism in a patient with infective endocarditis; only four similar cases have been reported in the literature.

## Introduction

Embolization is one of the main complications of infective endocarditis [1]. In left-sided endocarditis, the most frequent site of embolism is the brain, with other sites being spleen, liver, skin, iliac, and mesenteric arteries [1]. Paradoxical embolism is defined by embolic events happening via an intracardiac communication [2]. We described a case of aortic valve endocarditis with paradoxical pulmonary embolism in a patient with interventricular communication.

## Case presentation

**Figure 1:**
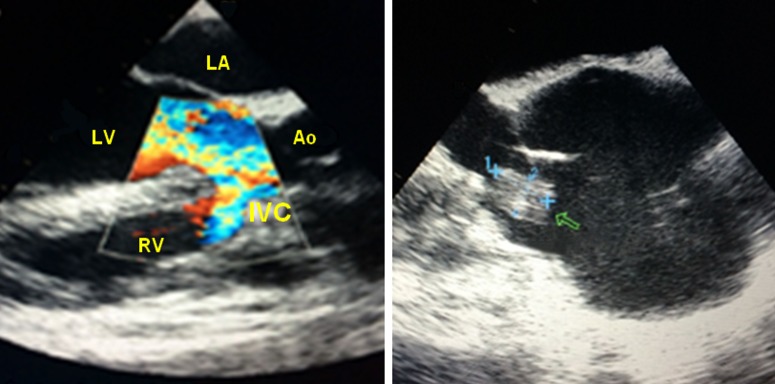
Transesophageal echocardiogram showing aortic regurgitation towards the right ventricle through an interventricular communication, and aortic valve vegetation (arrow). Ao: Aorta; IVC: Interventricular communication; LA: Left atrium; LV: Left ventricle; RV: Right ventricle.

A 35-year-old male with a past medical record of interventricular communication presented with progressive dyspnea for one month. He was admitted to the emergency department with vomiting and fever for the past five days. At physical examination, the patient exhibited tachycardia (122 bpm), blood pressure of 110/70 mmHg and an aortic systo-diastolic murmur, grade IV/VI. Chest X-ray revealed bilateral nodular infiltrates and laboratory exams showed the increase of nitrogenous compounds and metabolic acidosis. Transthoracic echocardiogram revealed left ventricular enlargement and aortic insufficiency, but no signs of endocarditis. Therefore, a transesophageal echocardiogram was performed (Figure 1), showing aortic valve vegetation, ascending aorta dilatation, aortic regurgitation, and the interventricular communication. Blood cultures were drawn and empirical treatment for infective endocarditis with Meropenem and Linezolid initiated. Cultures turned out positive for oxacillin-sensitive *Staphylococcus aureus*.

**Figure 2:**
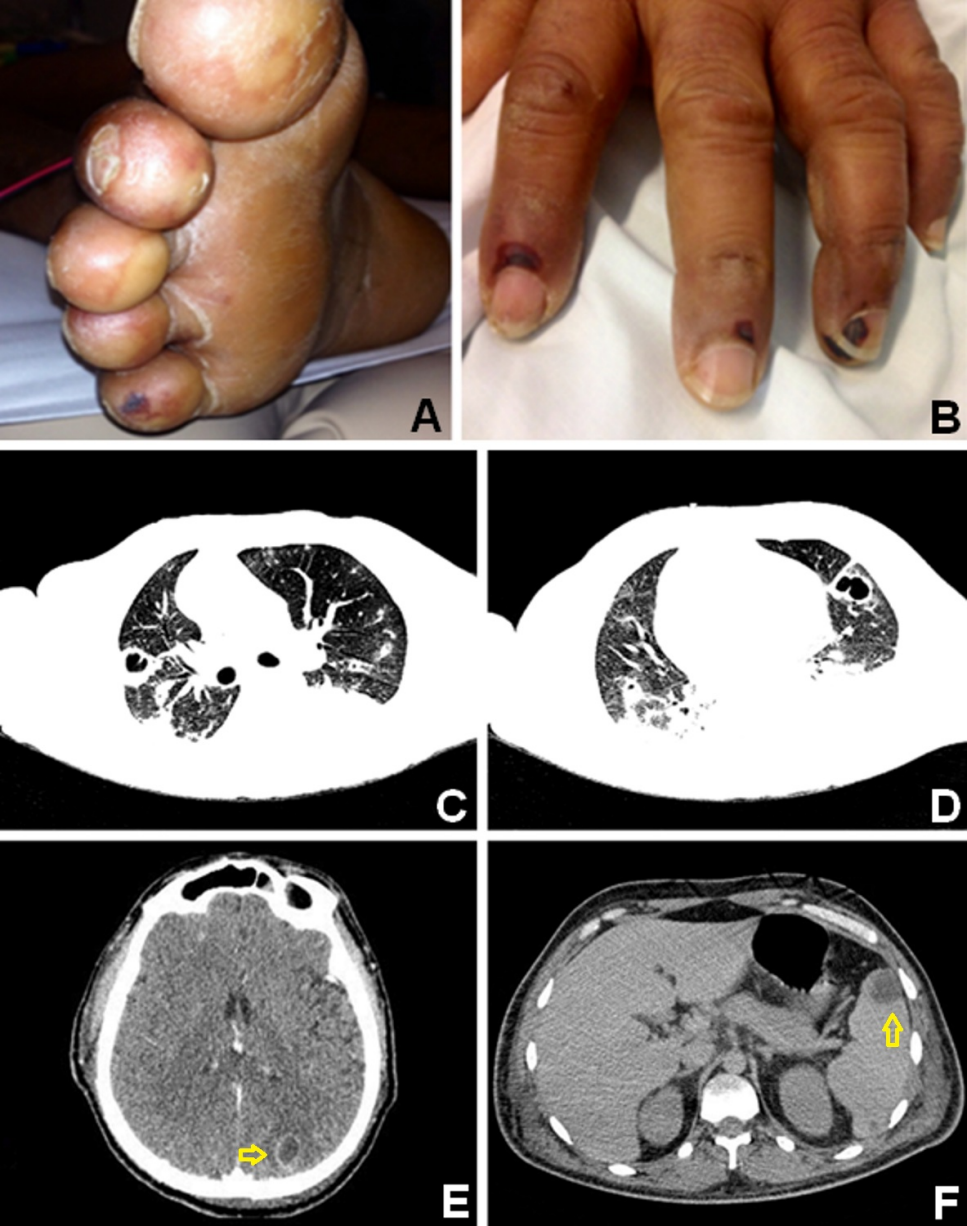
Evidence of peripheral (A and B) embolization from the aortic valve vegetation. Computed tomography scans show bilateral pulmonary (C and D), as well as cerebral (E) and splenic (F) embolisms (arrows).

The patient developed signs of peripheral embolization (Figure 2A-2B). Computed tomography (CT) scans showed evidence of lungs, brain, and splenic emboli (Figure 2C-2F). Cranial tomography revealed 2-3 cm hypodense cortico-subcortical areas at the left parieto-occipital and right occipital regions, consistent with embolic infarctions. Abdominal tomography revealed peripheral hypodense areas in the spleen and a subcapsular hypodense area at the inferior pole of the right kidney, both images consistent with ischemic lesions.

Contrast-enhanced CT of the chest revealed subpleural cuneiform consolidation areas, some with central excavation, findings consistent with septic embolism. Moderate left pleural effusion was also seen.

The patient was submitted to cardiac surgery. During the procedure, a vegetation at the ventricular side of the aortic valve and an abscess along the aortic ring were found. Extracorporeal circulation was used for 3 hours and 10 minutes and multiple blood transfusions were taken. The patient arrived at the intensive care unit hemodynamically unstable under high doses of noradrenalin and dobutamine. Unfortunately, the patient died at the fifth post-operatory day due to refractory shock.

## Discussion

Septic pulmonary emboli are most commonly found in cases of right-sided infective endocarditis [1]. Although intracardiac shunts, including ventricular septal defects, have a certain risk for paradoxical embolism [2], cases of aortic valve endocarditis with paradoxical embolization through interventricular communications are rarely described in the literature.

Only four similar cases have been reported [3-6]. In all cases, the lungs were the most significant site of embolism. In one of them [3], pulmonary embolism was associated with embolization to the popliteal artery. In two of the reported cases [3,4], the site of paradoxical passage was a congenital interventricular communication, similarly to our case. The remaining two cases had embolization through acquired communications, one through an arteriovenous fistula for hemodialysis [5], and the other between the ventricles caused by the endocarditis [6]. Similar to the latter [6] and unlike the other cases [3,4], the patient we described died likely due to the presence of significant aortic insufficiency at presentation and occurrence of multiple embolisms to different organs during hospitalization.

## Conclusions

This report describes a rare case of aortic valve infective endocarditis with paradoxical pulmonary embolism due to interventricular communication.
